# Proteomic identification of *Mycobacterium tuberculosis* glycoproteins as potential biomarkers for active tuberculosis: Development of a prototype competitive ELISA for sputum antigen detection

**DOI:** 10.1016/j.bbrep.2026.102867

**Published:** 2026-11-05

**Authors:** Shamsi Yari, Seyed Davar Siadat, Fatemeh Yari, Abolfazl Fateh, Alireza Hadizadeh Tasbiti

**Affiliations:** a Tuberculosis and Pulmonary Research Department, Pasteur Institute of Iran, Tehran, Iran; b Microbiology Research Center (MRC), Pasteur Institute of Iran, Tehran, Iran; c Blood Transfusion Research Center, High Institute for Research and Education in Transfusion, Tehran, Iran

**Keywords:** Affinity chromatography, LC-Mass spectrometry, Competitive ELISA, Glycoproteins, Proteomics

## Abstract

**Background:**

Protein glycosylation can influence the antigenicity of *Mycobacterium tuberculosis* (MTB) proteins, but glycoprotein antigens remain little explored for the development of antigen-detection assays for tuberculosis (TB). Here, MTB glycoproteins were enriched and identified by proteomics, and a prototype competitive enzyme-linked immunosorbent assay (ELISA) was developed for detection of TB-associated antigenic material in sputum.

**Methods:**

Glycoproteins from MTB strain TB-1039 were enriched by concanavalin A (ConA) affinity chromatography and characterized by SDS-PAGE, periodic acid–Schiff (PAS) staining, and liquid chromatography–mass spectrometry (LC-MS). GlycoPP and the Immune Epitope Database (IEDB) were used to predict N- and O-glycosylation sites and linear B-cell epitopes, respectively. The purified glycoproteins were conjugated to horseradish peroxidase (HRP), and antibodies purified from pooled sera of 10 culture-confirmed active-TB patients were used to develop the assay. The assay was subsequently evaluated using 206 sputum specimens collected from 132 patients with culture-confirmed active TB and 74 culture-negative comparator participants without active TB.

**Results:**

Thirteen glycoproteins (Rv3873, Rv0934, Rv1078, Rv0954, Rv0173, Rv0583c, Rv1270c, Rv1543, Rv0341, Rv1860, Rv2560, Rv0174, and Rv0838) were selected according to predicted glycosylation and epitope compatibility. Six candidates (Rv0954, Rv0174, Rv1078, Rv0838, Rv0583c, and Rv1860) exceeded 70% compatibility. The competitive ELISA yielded an apparent area under the receiver operating characteristic (ROC) curve of 0.951 (95% CI 0.92–0.98; *P* < 0.001). Using a cut-off of ≤0.65 chosen on the same specimens, apparent sensitivity was 90.15% (95% CI, 83.7–94.7%) and apparent specificity was 93.24% (95% CI, 84.9–97.8%).

**Conclusion:**

ConA enrichment, LC-MS identification, and in silico analysis enabled the prioritization of MTB glycoprotein candidates and supported development of a prototype competitive ELISA for TB-associated antigen detection in sputum. These preliminary, single-center findings provide a basis for further optimization, but independent validation in larger cohorts, including appropriate disease-control groups, is required before clinical application.

## Highlights

• ConA enrichment and LC-MS identified *Mycobacterium tuberculosis* glycoproteins.
• Thirteen glycoproteins were prioritized by predicted glycosylation and epitopes.
• Six candidates showed >70% glycosylation-epitope compatibility.
• A prototype competitive ELISA was built with affinity-purified antibodies.
• Apparent AUC was 0.951 in 206 specimens; independent validation is required.

## 1 Introduction

Tuberculosis (TB) remains a major global public health challenge, affecting millions each year [1]. According to the World Health Organization (WHO) Global TB report 2025, 8.3 million people were newly diagnosed with TB and accessed treatment in 2024, while important gaps remain in access to rapid diagnostic testing. Rapid and accurate diagnosis is central to timely treatment and TB control [2].

Xpert MTB/RIF Ultra and Xpert MTB/RIF are WHO-recommended nucleic acid amplification tests that can rapidly identify MTB complex and rifampicin resistance in respiratory samples. Their speed and potential for near-patient use are important advantages; however, implementation depends on molecular instrumentation, consumables, and supporting infrastructure, which may restrict their use in resource-limited settings [3].

Protein glycosylation is an important post-translational modification that can influence protein structure, localization, stability, antigenicity, and interactions with host immune components. [4]. MTB contains numerous glycosylated and glycolipoproteins, and previous proteomic work identified 293 candidate glycoproteins using ConA enrichment, LC-MS, GlycoPP, and IEDB analyses [5], [6], [7]. The present study extends that work by prioritizing a subset of glycoproteins and developing a prototype antibody-based assay for TB-associated antigen in sputum.

Thirteen candidate proteins were selected for further analysis on the basis of predicted glycosylation and the apparent overlap between glycosylation sites and predicted antigenic determinants. Their predicted linear B-cell epitopes were evaluated using the IEDB BepiPred-2.0 resource. The purified glycoprotein preparation was subsequently used to develop a prototype antibody-based competitive ELISA for clinical sputum specimens [5].

Competitive enzyme-linked immunosorbent assay (ELISA) is based on competition between an analyte in the test sample and a labeled antigen for a limited amount of antibody. Increasing concentrations of the sample antigen reduce binding of the labeled antigen and therefore decrease the measured signal [8]. In the present study, this format was adapted to evaluate whether antibodies raised against MTB glycoproteins could support detection of TB-associated antigen in sputum.

The aim of this study was to develop and characterize a prototype glycoprotein-based competitive ELISA for the detection of TB-associated antigen in sputum and to obtain a preliminary estimate of its apparent discriminatory performance using culture-confirmed clinical specimens. Specifically, we enriched and identified MTB glycoproteins, prioritized candidates by predicted glycosylation and epitope compatibility, prepared glycoprotein-specific antibodies, optimized the assay conditions, and estimated the apparent sensitivity, specificity, and overall discriminatory ability using receiver operating characteristic (ROC) curve analysis.

## 2 Material and methods

### 2.1 Study settings

The study was conducted in the Tuberculosis and Pulmonary Research Department of the Pasteur Institute of Iran, Tehran. A total of 206 adult participants were included. Sputum specimens were processed using the same laboratory workflow, and the ELISA plate coating, incubation, washing, and absorbance measurements were performed by laboratory technologists who were blinded to the clinical and microbiological classification of each specimen and had access only to coded specimen numbers. ROC analysis was performed by a statistician using group-coded, anonymized data. Assignment of specimen codes and final unblinding were performed by the principal investigator after database lock. In addition, sera from 10 adult patients with culture-confirmed active TB were collected and pooled specifically for purification of anti-glycoprotein antibodies. Written informed consent was obtained from all participants before enrollment in the study. The protocol complied with the 1975 Declaration of Helsinki and local requirements and was approved by the Ethics Committee of the Pasteur Institute of Iran (Approval ID: IR.PII.REC.1401.029).

### 2.2 Experimental replication

The diagnostic evaluation included 206 sputum specimens collected from individual participants, comprising 132 patients with active-TB and 74 comparator participants. Each participant was considered an independent biological unit for the diagnostic accuracy analysis. Where applicable, ELISA measurements were performed in duplicate wells as technical replicates. Duplicate measurements were not treated as independent biological replicates. No additional independent biological replication of the specimens was performed.

### 2.3 Sample size considerations

The sample size was determined based on preliminary data from previous TB investigations. A power analysis was performed to achieve at least 80% statistical power to detect a significant difference in diagnostic performance between participants with active-TB and comparator participants, using a two-sided significance level of 0.05. This analysis yielded a required sample size of 206 participants, taking into account the expected prevalence of TB and anticipated response rates in the study population. As this was a single-center proof-of-concept study, the estimated diagnostic performance should be regarded as an apparent (internal) estimate, as the cutoff was derived and evaluated using the same specimens and therefore requires independent external validation.

### 2.4 Study Groups and Eligibility

The study population comprised two groups: (i) the active-TB group (n = 132), consisting of patients with clinical and radiographic findings suggestive of active pulmonary TB and microbiological confirmation of MTB by sputum culture; and (ii) the comparator group (n = 74), consisting of individuals with no evidence of clinically active TB, as indicated by the absence of compatible clinical and radiographic findings and negative sputum smear and culture results. Individuals with smear-positive but culture-negative specimens were not included in the active-TB group. The comparator group also included individuals with a history of previous TB, stable radiographic abnormalities, and/or a positive tuberculin skin test, provided that there was no evidence of active disease at enrollment. These group definitions were applied consistently throughout the manuscript, including the tables and figures (Table 1).

**Table 1:** Demographic data and description of patients studied.

Characteristic	Subgroup/Value	Total N (%)	Active-TB group n (%)	Comparator group n (%)	*P*-value
**Age (years)**	Mean ± SD	43.27 ± 12.29	43.80 ± 12.10	42.30 ± 12.60	0.41
	Maximum	79.00	-	-	-
	Minimum	19.00	-	-	-
	Range	60.00	-	-	-
	< 20	2 (1.0%)	1 (0.8%)	1 (1.4%)	0.91
	20 – 40	92 (45.0%)	60 (45.5%)	32 (43.2%)	
	40 – 60	90 (43.0%)	56 (42.4%)	34 (45.9%)	
	> 60	22 (11.0%)	15 (11.4%)	7 (9.5%)	
**Sex**	Male	96 (46.6%)	63 (47.7%)	33 (44.6%)	0.67
	Female	110 (53.4%)	69 (52.3%)	41 (55.4%)	
	Total	206 (100.0%)	132 (100.0%)	74 (100.0%)	
**Study groups**^a^	Culture-positive	132 (64.1%)	132 (64.1%)	0 (0.0%)	–
	Culture-negative	74 (35.9%)	0 (0.0%)	74 (35.9%)	–

a Culture is considered the gold standard for confirming TB disease in the study groups. A positive culture for *Mycobacterium tuberculosis* confirms the diagnosis of active TB disease. Statistical analysis: Age (a continuous variable) was compared between groups using Welch's independent-samples *t*-test. Age-category and sex distributions were compared using Pearson's chi-square test. The comparator group consisted of culture-negative participants without evidence of clinically active TB.

Eligibility for the active-TB group was based on compatible clinical findings, suggestive chest radiographic or computed tomography (CT) abnormalities, and microbiological confirmation. Clinical features included symptoms consistent with pulmonary infection, such as cough, fever, and weight loss. Sputum specimens were required to be of adequate quality, characterized by few squamous epithelial cells and a high number of white blood cells. Patients were excluded from the TB group if their specimens were of poor quality, including samples containing excessive saliva or numerous epithelial cells, or if they had a recent upper respiratory tract infection or other conditions that could interfere with the clinical assessment of TB [9]. Age and sex were recorded as demographic characteristics.

Sputum specimens were examined by Ziehl–Neelsen microscopy and cultured on Lowenstein–Jensen medium following N-acetyl-l-cysteine–sodium hydroxide (NALC–NaOH) processing [10]. Isolation of *M. tuberculosis* by culture was required for classification as active TB; smear positivity alone was not considered sufficient for inclusion in the TB group.

Although inclusion of disease-control groups, such as patients with non-tuberculous mycobacterial (NTM) infection or bacterial pneumonia, would have provided a more comprehensive assessment of the assay's ability to distinguish active TB from other respiratory conditions, such groups were not included in the present study. Their inclusion in future validation studies will be important for further establishing the specificity and clinical utility of the assay.

### 2.5 TB culture and protein precipitation

MTB strain TB-1039, originally isolated from a patient and maintained in the TB strain bank of the Pasteur Institute of Iran, Tehran, Iran, was cultured on Proskauer–Beck medium at 37 °C for four weeks. The cells were harvested by centrifugation at 3000 × g for 30 min, washed with phosphate-buffered saline (PBS; pH 7.3), and transferred to a purification buffer containing 1 mM phenylmethylsulfonyl fluoride (PMSF), 20 mM EDTA, 10% glycerol, 40 mM Tris, 0.5% Triton X-114, 1 μg/mL DNase, and 0.02% sodium azide. The suspension was sonicated and centrifuged, followed by dialysis against PBS. Total protein concentration was determined using the Bradford assay [11].

SDS-PAGE was performed using a Mini-PROTEAN Tetra Cell system (Bio-Rad) with 10% polyacrylamide gels, as previously described [12]. Following electrophoresis, the gels were stained using the periodic acid–Schiff (PAS) method to visualize glycoprotein-containing bands [13]. The original single-panel gel is incorporated as panel A of the consolidated Fig. 1.

**Fig. 1:**
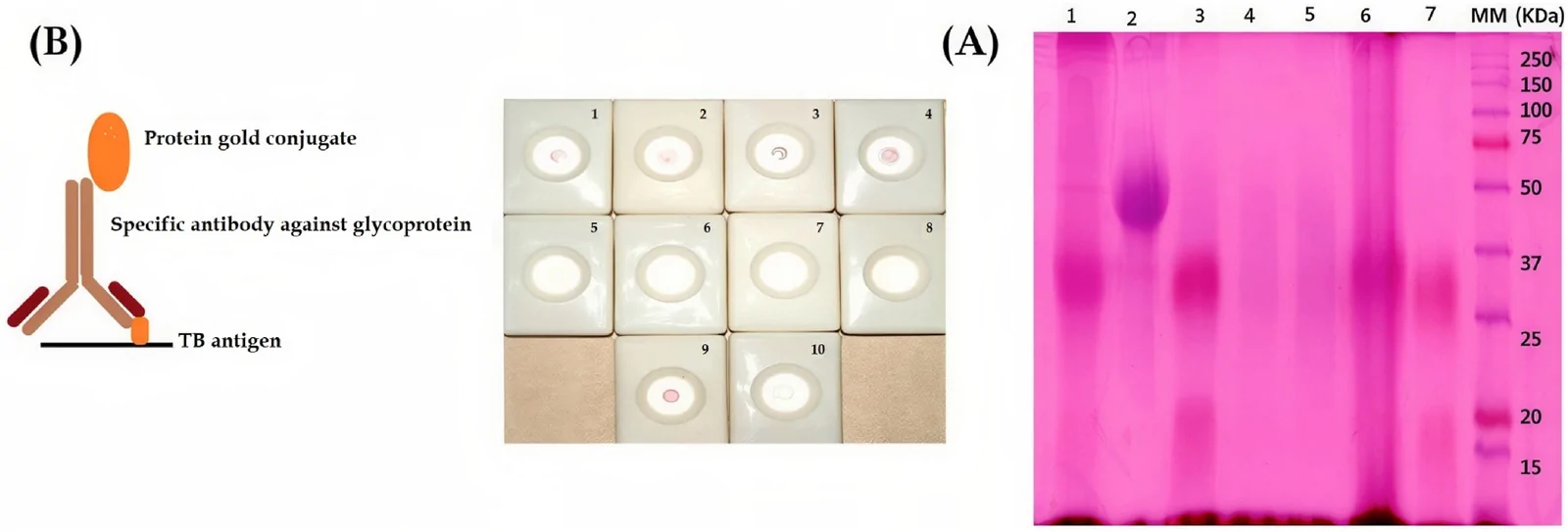
Glycoprotein characterization and immunochromatographic evaluation. **(A)** Representative periodic acid–Schiff (PAS)-stained SDS-PAGE of fractions obtained after ConA affinity chromatography. Lanes 1–7 represent protein fractions/peaks; lane 8 contains molecular-weight markers (250, 150, 100, 75, 50, 37, 25, 20, and 15 kDa). **(B)** Schematic representation and representative immunochromatographic reactions. Cartridges 1–4 show positive reactions with a central red-pink signal; cartridges 5–8 show negative reactions. Cartridge 9 is the BCG positive control and cartridge 10 is the PBS negative control. ConA, concanavalin A; BCG, Bacillus Calmette–Guérin; PBS, phosphate-buffered saline.

### 2.6 Concanavalin A affinity chromatography

Glycoproteins were enriched by Concanavalin A (ConA) affinity chromatography using a 30-cm-long column with an internal diameter of 1.8 cm, packed with 5 mL of ConA–Sepharose 4B (Sigma, USA) resin. The column was equilibrated with ConA buffer consisting of 700 mL of distilled water, 100 mL of 1 M Tris-HCl (pH 7.2–7.4), 5 mL of 1 M MgCl_2_, 5 mL of 1 M CaCl_2_, and 29.2 g of NaCl (0.5 M), with distilled water added to a final volume of 1 L (pH 7.2).

A 500-μL aliquot of the total protein preparation was applied to the equilibrated column and incubated for 1 h to allow glycoproteins to bind to the ConA resin. Bound glycoproteins were subsequently eluted using α-methyl-d-mannopyranoside at concentrations ranging from 0.2 to 1 M, which competes with glycan moieties for binding to ConA. Fractions collected during elution were monitored by measuring absorbance at 280 nm.

The eluted fractions were further analyzed by SDS-PAGE and periodic acid–Schiff (PAS) staining to detect and characterize glycoproteins based on their electrophoretic mobility (Fig. 1A) [11], [14], [15], [16], [17], [18]. The extracted glycoproteins were subsequently characterized by liquid chromatography–mass spectrometry (LC–MS). Proteomic data were analyzed to identify candidate glycoproteins, and the performance of the competitive ELISA was evaluated using receiver operating characteristic (ROC) curve analysis.

### 2.7 LC- mass spectrometry and data analysis

Protein spots of interest were excised from the gel into approximately 1 × 1 mm pieces and submitted to PhenoSwitch Bioscience (Sherbrooke, Canada) for liquid chromatography–tandem mass spectrometry (LC–MS/MS) analysis. The gel pieces were dehydrated using 50 mM Tris buffer and acetonitrile, followed by rehydration with 10 mM dithiothreitol (DTT) and incubation at 65 °C for 15 min. The proteins were then alkylated with 15 mM iodoacetamide at room temperature for 30 min. After dehydration to remove excess reagents, the gel pieces were rehydrated in 50 mM Tris buffer (pH 8.0) containing 1 μg of trypsin and incubated at 37 °C with shaking to facilitate enzymatic digestion. The resulting peptides were purified by reversed-phase solid-phase extraction before LC–MS/MS analysis.

Mass spectrometric analysis was performed using an AB SCIEX TripleTOF 5600 mass spectrometer (AB SCIEX, Foster City, CA, USA). Instrument control and data acquisition were carried out using Analyst TF software (version 1.7). The ion-source voltage was set to 5.2 kV, and the source temperature was maintained at 225 °C. Peptides were separated using a reversed-phase HALO C18-ES column with a reported particle size of 2.7 μm. The internal diameter of the column was not available to the authors. The column temperature was maintained at 50 °C, and samples were loaded through a 5 μL injection loop. Chromatographic separation was performed using a mobile-phase gradient comprising solvent A (water containing 3% dimethyl sulfoxide [DMSO] and 0.2% formic acid) and solvent B (ethanol containing 3% DMSO and 0.2% [v/v] formic acid), at a flow rate of 10 μL/min.

Protein identification was performed using ProteinPilot™ software (version 4.0; SCIEX). The protein sequence database used, its taxonomic scope, the peptide- and protein-level identification criteria, and the false discovery rate (FDR) threshold applied in the ProteinPilot search were not available to the authors and therefore could not be reported. This limitation is acknowledged in Section 4.1. The amino acid sequences of the identified proteins were retrieved, and sequence similarity searches were conducted using the protein Basic Local Alignment Search Tool (BLASTp) provided by the National Center for Biotechnology Information (NCBI; https://www.ncbi.nlm.nih.gov/). The functional classification of the identified proteins was determined using the MycoBrowser database (https://mycobrowser.epfl.ch/genes) [5].

The identified proteins were subsequently evaluated for potential post-translational modifications using the GlycoPP web server (http://www.imtech.res.in/raghava/glycopp/; version 1.0, accessed in 2011), which predicts potential N- and O-glycosylation sites in prokaryotic protein sequences. Candidate membrane- and surface-associated proteins with higher predicted glycosylation scores were further analyzed using the Immune Epitope Database (IEDB, http://www.iedb.org; accessed in 2017), including the BepiPred-2.0 tool, to predict potential linear B-cell epitopes [18], [19].

### 2.8 Peroxidase-labeled glycoprotein

First, 5 mg of peroxidase (POD; Roche, Germany) was dissolved in 1 mL of 0.3 M sodium bicarbonate (NaHCO_3_; pH 8.1), and 50 μL of 1% fluorodinitrobenzene (FDNB; Sigma) in %1 ethanol was added. The mixture was gently stirred for 1 h at room temperature. Subsequently, 1 mL of 0.1 M sodium periodate (NaIO_4_) solution was added, and the mixture was gently stirred for an additional 30 min at room temperature. Sodium periodate in an aqueous medium was used to oxidize the carbohydrate moieties of HRP, generating aldehyde groups for subsequent conjugation. The reaction was stopped by adding 1 mL of 0.5 M ethylene glycol in distilled water, followed by gentle stirring at room temperature. After 1 h, the activated horseradish peroxidase (HRPO) was dialyzed overnight against 0.01 M sodium carbonate-bicarbonate buffer at 4 °C.

Next, 20 mg of purified glycoprotein (the ConA-enriched glycoprotein fraction) was prepared in 0.01 M sodium carbonate-bicarbonate buffer (pH 9.5). The glycoprotein solution was then added to the activated HRPO solution and gently mixed for 2 h at room temperature. Freshly prepared 1% potassium borohydride (KBH_4_; 0.3 mL) was added to the conjugation mixture, which was allowed to stand for 1 h at room temperature with occasional mixing. Finally, the reaction mixture was adjusted to pH 7 using HCl and dialyzed against PBS at 4 °C. The RZ value was then determined as the ratio of absorbance at 403 nm to that at 280 nm (A_403_/A_280_) measured using a UV–Vis spectrophotometer (Jenway 6305, Fisher Scientific, Loughborough, UK) [20], [21], [22].

### 2.9 Purification of specific antibody against MTB glycoprotein

Prior to the immunochromatographic and ELISA assays, affinity chromatography was used to purify specific antibodies against MTB glycoproteins. To obtain sera from patients with active-TB (active-TB group), blood samples were collected from 10 adult subjects whose TB diagnosis had been previously confirmed by culture, as described in Section 2.4 (Study Groups and Eligibility). Sera from these 10 patients were pooled before affinity purification; individual sera were not processed separately.

First, the total purified glycoprotein was conjugated to CNBr-activated Sepharose 4B (Sigma, USA), packed into an affinity column (25 × 5 cm), and equilibrated with 0.1 M Tris buffer. In the next step, 2.5 mL of pooled serum obtained from the 10 TB patients was applied to the column. After washing the column with 0.1 M Tris buffer, specific antibodies against the glycoproteins were eluted by adding 0.1 M glycine buffer (pH 2.8). The yield of purified antibody was 0.9 mg/mL, and the antibody concentration was determined by measuring absorbance at 280 nm (A280). Finally, the specificity and sensitivity of the purified antibodies were determined using an immunochromatographic method [23], [24], [25].

Because the purified antibodies and HRP–glycoprotein conjugate were prepared in-house and are not commercially available, other laboratories can reproduce them using the protocols described above. Aliquots may also be obtained from the corresponding authors upon reasonable request, subject to the applicable institutional material-transfer procedures.

### 2.10 Immunochromatographic method

Sputum samples from 206 adult participants (132 active-TB and 74 comparator) were decontaminated using the NALC-NaOH method. A 200 μL aliquot of each prepared sputum specimen was added to the reaction cartridge and allowed to absorb completely. After washing with PBS containing Tween 20, the purified antibody against the glycoprotein was added to the cartridge. Following a second washing step, 200 μL of the protein-gold conjugate (40 nm, British Biocell, UK) was added, followed by a final washing step to remove unbound conjugate.

A positive immunochromatographic reaction was defined by development of a red-pink signal in the central region of the cartridge, whereas a negative reaction showed no central color. PBS and BCG (whole bacteria) were used as negative and positive controls, respectively. Representative results are shown in Fig. 1B.

### 2.11 Competitive ELISA

#### 2.11.1 Sputum sample procedures

Before performing the ELISA assays, the NALC–NaOH method was used for sputum decontamination. Sputum specimens were processed using a NaOH–NALC decontamination procedure based on the method described by Kent and Kubica [26], with modifications in accordance with the laboratory's established protocol. Briefly, an equal volume of NaOH–NALC processing solution was added to each sputum specimen, and the mixture was vortexed for 1 min to achieve a final NaOH concentration of 1%. The samples were then incubated at room temperature for 15 min to allow NALC to liquefy the mucus. Phosphate buffer (0.0067 M, pH 6.8) was subsequently added to adjust the final volume to 50 mL. The specimens were centrifuged at 3000 × g for 15 min, after which the supernatant was discarded and the pellet containing concentrated microbial cells was retained for subsequent analyses. The processed specimens were used for culture and diagnostic testing, including the ELISA procedure. Each specimen was processed in duplicate where applicable, and ELISA measurements were performed in duplicate wells as technical replicates; these replicate measurements were not considered independent biological replicates.

#### 2.11.2 Preparation of ELISA plate for competitive ELISA

Specific antibodies (anti-glycoprotein antibodies) purified from TB patient sera were dialyzed against carbonate–bicarbonate buffer (15 mM Na_2_CO_3_, 35 mM NaHCO_3_, pH 9.6–9.7). Purified anti-glycoprotein antibodies were used to coat the ELISA plates at a concentration of 8.6 μg/mL, with 100 μL of antibody solution added to each well. The plates were incubated overnight to allow passive antibody adsorption. After incubation, the excess antibody solution was removed, and each well was blocked with 200 μL of blocking buffer consisting of 1% BSA in PBS containing 0.05% sodium azide. The plates were then incubated overnight under refrigeration. Immediately before use in the assay, the HRP–glycoprotein conjugate was diluted 1:32 in PBS to prepare the working solution.

#### 2.11.3 Titration of the peroxidase-labeled glycoprotein

The blocking buffer was removed by a simple washing step in which the wells were flooded and emptied using PBS containing Tween 20. Subsequently, HRP-conjugated glycoproteins were added to target the antigenic sites on the solid-phase antibodies. During incubation, the glycoproteins bound to the antibodies, followed by another washing step to remove any unbound peroxidase-labeled glycoprotein.

Next, the substrate o-phenylenediamine (OPD; Sigma) was added. The substrate reacted with the HRP enzyme associated with the bound glycoproteins, resulting in a color reaction. The reaction was allowed to proceed for a defined period and was then stopped by adding sulfuric acid to alter the pH. Finally, the color intensity was quantified using an ELISA plate reader (Stat Fax‐2100, Awareness Technology Inc., USA) by measuring absorbance at 492 nm.

#### 2.11.4 Patient analysis using competitive ELISA

The blocking buffer was removed by a simple washing step in which the wells were flooded and emptied using PBS containing Tween 20. A volume of 100 μL of each processed sputum specimen was added to duplicate wells and incubated for 2 h at room temperature to allow the antibodies to bind to the antigens. Subsequently, the HRP–glycoprotein conjugate solution was added to each well, and the plates were incubated for an additional 1 h at room temperature.

The HRP–glycoprotein conjugate solution was then discarded, and the wells were washed three times with wash buffer (0.05% Tween 20 in PBS). The substrate solution was subsequently added to the wells and incubated for 30 min at room temperature in the dark. The OPD substrate was used for all patient specimens and controls, following the same procedure as that used for conjugate titration. The substrate was added to the wells, and the reaction was allowed to proceed for 30 min at room temperature in the dark. The reaction was then stopped by adding sulfuric acid (H_2_SO_4_), and absorbance was measured at 492 nm using an ELISA plate reader (Stat Fax‐2100, Awareness Technology Inc., USA). The assay was interpreted qualitatively by comparing the mean optical density (OD_492_) of duplicate wells for each specimen with the predefined cut-off value. Specimens with an OD_492_ ≤ 0.65 were classified as positive, whereas those with values above this threshold were classified as negative. Antigen concentrations were not quantified [27], [28], [29].

#### 2.11.5 Controls and run acceptance criteria

For quality control of the competitive ELISA, PBS was included on each plate as the negative control, while a previously characterized sputum specimen from a patient with confirmed active-TB served as the positive control. Both controls were processed using the same procedure as the clinical specimens, and all controls and patient samples were tested in duplicate.

An ELISA run was considered valid if the PBS negative control produced the expected reference signal, the TB-positive sputum control showed the expected inhibitory response relative to the negative control, and the duplicate wells demonstrated acceptable agreement. Because this was an exploratory prototype assay, formal numerical acceptance criteria (for example, a defined optical-density range for the PBS negative control, a minimum percentage inhibition for the TB-positive sputum control, or a maximum coefficient of variation between duplicate wells) were not predefined; these three conditions were therefore assessed qualitatively by the operators. Plates were rejected and the assay repeated if either control failed to produce the expected response or if substantial variation occurred between duplicate control wells. Only results from valid assay runs were included in the final analysis.

#### 2.11.6 Calibration curve

The HRP–glycoprotein conjugate was titrated under the optimized assay conditions using anti-glycoprotein antibodies. Serial dilutions of the conjugate (1:1 to 1:128) were tested, followed by the addition of o-phenylenediamine (OPD; Sigma, USA) as the substrate. Absorbance was measured at 492 nm, and the mean absorbance values obtained from the serial dilutions were plotted to generate the calibration curve (conjugate titration) (Fig. 2A). The assay was used as a qualitative, cut-off-based test; this curve documents the titration of the conjugate and is not an antigen standard curve.

**Fig. 2:**
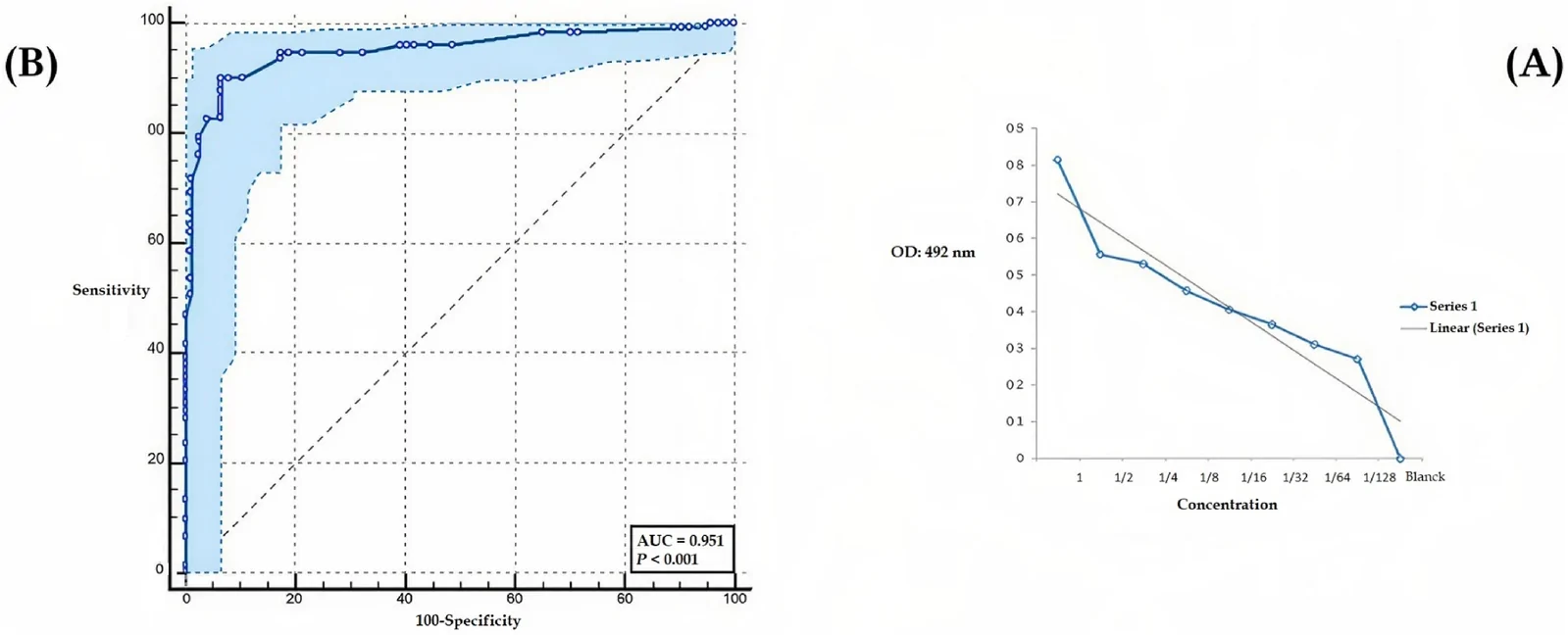
Calibration and apparent diagnostic performance of the competitive ELISA. (A) Calibration curve obtained from serial dilutions of the HRP–glycoprotein conjugate (1:1 to 1:128) measured at 492 nm under the optimized assay conditions. Each dilution was measured in duplicate (technical replicates), and mean absorbance values were used for the curve. (B) Receiver operating characteristic (ROC) analysis of the competitive ELISA in 206 sputum specimens (132 active-TB and 74 comparator). The apparent AUC was 0.951 (95% CI, 0.92–0.98; *P* < 0.001 versus 0.5). The cut-off (≤0.65; criterion associated with the highest Youden index) gave an apparent sensitivity of 90.15% (95% CI, 83.7–94.7%) and an apparent specificity of 93.24% (95% CI, 84.9–97.8%); it was selected and evaluated on the same specimens. The shaded area shows the 95% confidence bounds of the ROC curve. ROC analysis was performed using MedCalc® Statistical Software version 22.018. HRP, horseradish peroxidase; TB, tuberculosis; ROC, receiver operating characteristic; AUC, area under the curve.

### 2.12 Data analysis

Statistical analyses were performed using MedCalc® Statistical Software version 22.018 (MedCalc Software Ltd, Ostend, Belgium; 2024). ROC curve analysis was used to determine cut-off value for discriminating active-TB from comparator specimens [30]. The cut-off value was selected to maximize the Youden index (J = sensitivity + specificity − 1) [31]. The area under the receiver operating characteristic curve (AUC) was compared with the null value of 0.5 using a z-test. The 95% confidence interval (CI) for the AUC was calculated as AUC ± 1.96 × standard error (SE), with the SE estimated using the method of Hanley and McNeil [32], based on the AUC and the sizes of the two study groups. Because the cut-off was selected and evaluated using the same specimens, the diagnostic performance measures represent apparent (resubstitution) estimates and may be optimistic. Age was compared between the active-TB and comparator groups using Welch's independent-samples *t*-test, and the age-category and sex distributions (Table 1) were compared using Pearson's chi-square (χ^2^) test. A *P* value < 0.05 was considered statistically significant. Sensitivity and specificity were calculated using standard diagnostic-test definitions [33]. Predictive values and efficiency were calculated in the same way. Exact (Clopper–Pearson) 95% confidence intervals were calculated for sensitivity, specificity, predictive values, and efficiency [34]. All 206 enrolled specimens were included in the analysis; no specimen was excluded because of a missing or invalid result, and results were classified as positive or negative without an indeterminate category.

In the competitive binding assay, the competitor antigen competes with the target analyte, such as TB antigen in sputum, for binding to the primary antibody. As the concentration of the competitor increases, the binding of the target antigen decreases, resulting in a lower absorbance signal. Thus, the degree of signal inhibition is related to the concentration of the competitor and can be used to estimate the amount of the target analyte present in the sample.

### 2.13 Replication and experimental unit

Each specimen was assayed in duplicate wells (technical replicates), and the mean absorbance was used. The independent experimental unit for the diagnostic evaluation was the individual participant (sputum specimens from 206 participants). Independent repeats of the complete assay on patient specimens (biological replicates) are not reported in this study.

## 3 Results

### 3.1 Glycoprotein identification and candidate selection

The identified MTB glycoproteins, together with their percentile compatibility, immunogenicity scores, predicted glycosylation percentages, and subcellular locations, are presented in Table 2, while predicted peptides based on residue scores obtained using BepiPred-2.0 through the IEDB analysis resource are presented in Table S1.

**Table 2:** Characteristics of glycoproteins (glycosylation prediction, immunogenicity, and location).

Gene (Protein)	Percentile Compatibility (%)	Immunogenicity score	Glycosylation (%)	Protein location
Rv3873, PPE family immunomodulatory, PPE68	60.0	>0.6	11.68	Cell wall and membrane
Rv0934, Phosphate-binding protein PstS	53	>0.6	9.0	membrane
Rv1078, Proline-rich antigen homolog, pra	87	>0.6	9.58	membrane
Rv0954, 34 kDa antigenic protein homolog	95	>0.6	13.86	membrane
Rv0173, Possible Mce-family lipoprotein LprK	50	3	5.4	Cell wall
Rv0583c, Probable conserved lipoprotein, LpqN	78	>0.6	11.84	membrane
Rv1270c, Lipoprotein LprA, LprA	60	>0.6	10.2	membrane
Rv1543, Uncharacterized oxidoreductase Rv1543	62	>0.6	6.16	membrane
Rv0341, Isoniazid-induced protein IniB	68	2	7.1	Cell wall
Rv1860, Alanine and proline-rich secreted protein, Apa	71.5	3	9.6	Cell wall
Rv2560, Uncharacterized, Probable proline and glycine rich transmembrane protein	62.5	4	7.4	membrane
Rv0174, Mce-family protein, Mce1F	87.5	2	6.21	Cell wall
Rv0838, D-alanyl-d-alanine dipeptidase, lpqR	85	>0.6	7.81	Cell wall

Six proteins (Rv0954, Rv0174, Rv1078, Rv0838, Rv0583c, and Rv1860) had a percentile compatibility above 70%.

Based on the prediction of N- and O-glycosylation sites in the identified glycoprotein sequences using the GlycoPP web server and the analysis of epitopes using IEDB, candidate glycoproteins were selected for further evaluation. Because the selection of appropriate threshold values is important for the stability of glycosylation-site prediction, performance was assessed across a range of score thresholds from −1.0 to 1.0, and optimal thresholds were identified for further analysis. Consequently, thirteen proteins (Rv3873, Rv0934, Rv1078, Rv0954, Rv0173, Rv0583c, Rv1270c, Rv1543, Rv0341, Rv1860, Rv2560, Rv0174, and Rv0838) showing high compatibility between predicted glycosylation sites and antigenic sequences, together with optimal threshold values, were selected (Table 2).

A high degree of compatibility was observed between glycosylation sites and the locations of predicted epitopes and antigenic determinants in the identified glycoproteins. These glycosylated amino acid residues were located within or in close proximity to predicted immunogenic epitopes. Among the 13 selected proteins, percentile compatibility ranged from 50% to 95%, and six (Rv1078, Rv0954, Rv0583c, Rv0174, Rv0838, and Rv1860) showed high percentile compatibility (>70%) (Table 2)**.**

### 3.2 Immunochromatographic evaluation of the purified antibody

The purified antibody was evaluated by immunochromatography using 206 sputum specimens (Fig. 1B). The assay yielded a sensitivity of 92.4% (122/132; 95% CI, 86.5–96.3%) and a specificity of 94.6% (70/74; 95% CI, 86.7–98.5%). The positive and negative predictive values were 96.8% (122/126; 95% CI, 92.1–99.1%) and 87.5% (70/80; 95% CI, 78.2–93.8%), respectively. Overall diagnostic efficiency was 93.2% (192/206; 95% CI, 88.9–96.2%) (Table 3). These results represent apparent performance estimates obtained from the same specimens used for evaluation, without an independent validation set.

**Table 3:** Apparent performance of the purified antibody in the immunochromatographic test (206 specimens).

Indicator	Value (%)	95% CI (%)	Calculation Formula (based on table data)
Efficiency	93.2%	88.9–96.2	(122 + 70)/(132 + 74)
Negative Predictive Value (NPV)	87.5%	78.2–93.8	70/(70 + 10)
Positive Predictive Value (PPV)	96.8%	92.1–99.1	122/(122 + 4)
Specificity	94.6%	86.7–98.5	70/(70 + 4)
Sensitivity	92.4%	86.5–96.3	122/(122 + 10)

Formula Guide (Definitions).

• **Sensitivity** = True Positive/(True Positive + False Negative).

• **Specificity** = True Negative/(True Negative + False Positive).

• **Positive Predictive Value (PPV)** = True Positive/(True Positive + False Positive).

• **Negative Predictive Value (NPV)** = True Negative/(True Negative + False Negative).

• **Efficiency** = (True Positive + True Negative)/Total Number of Cases.

### 3.3 HRP–glycoprotein conjugate and titration

The quality of the HRP–glycoprotein conjugate was evaluated using the RZ value, calculated as the ratio of absorbance at 403 nm to that at 280 nm (A_403_/A_280_). The RZ value reflects the heme content and purity of the peroxidase preparation. Highly purified peroxidase typically has an RZ value of approximately 3, whereas an RZ value of around 0.4 is considered acceptable for conjugates intended for use in immunoassays.

In this study, OD_403_ was 0.40 and OD_280_ was 0.857, giving an RZ value of 0.46. The conjugate was therefore considered suitable for subsequent assay development. Binding of the HRP–glycoprotein conjugate to antibody-coated wells produced a measurable colorimetric signal after substrate addition. The titration (calibration) curve obtained from serial dilutions of the conjugate is shown in Fig. 2A.

The binding and enzymatic activity of the HRP–glycoprotein conjugate were evaluated using an ELISA plate. In this assay, the glycoproteins bound to antibodies coated onto the plate. After washing to remove unbound material, the HRP–glycoprotein conjugate remained associated with the antibodies. Following the addition of the substrate, the enzymatic reaction produced a colorimetric signal, resulting in an increase in absorbance compared with the blank.

### 3.4 Competitive ELISA and ROC results

Sputum specimens were collected from 206 adult participants and evaluated using the competitive ELISA assay. Of these, 132 (64.1%) were classified as active-TB and 74 (35.9%) as comparator participants. The demographic characteristics of the study population are presented in Table 1. A chi-square test of independence showed no statistically significant association between sex and study group (χ^2^(1, N = 206) = 0.19, *P* = 0.67). Age did not differ significantly between the groups (43.80 ± 12.10 vs 42.30 ± 12.60 years; Welch's independent-samples *t*-test, *P* = 0.41), and the age-category distribution was also similar (χ^2^ test, *P* = 0.91).

ROC curve analysis included 206 participants (132 active-TB and 74 comparator participants) and was performed to assess the ability of the competitive ELISA to distinguish active TB from comparator specimens. The ROC curve plots the true-positive rate (sensitivity) against the false-positive rate (1 − specificity) across different assay thresholds. The apparent AUC was 0.951 (95% CI, 0.92–0.98), indicating strong discriminatory ability in this dataset. The AUC was significantly greater than 0.5 (z = 31.6, *P* < 0.001). The cut-off value that maximized the Youden index (J = 0.834) was ≤0.65. At this threshold, the assay yielded an apparent sensitivity of 90.15% (119/132; 95% CI, 83.7–94.7%) and an apparent specificity of 93.24% (69/74; 95% CI, 84.9–97.8%). Because the cut-off was selected and evaluated using the same specimens, these estimates may overstate the assay's diagnostic performance in independent populations and require validation in an external cohort. The corresponding ROC curve is shown in Fig. 2B.

## 4 Discussion

The diagnosis of active pulmonary TB relies mainly on microbiological and molecular methods. Although culture is an important reference method, its prolonged turnaround time can delay clinical decision-making, while molecular tests provide faster results but require specialized equipment and resources. These limitations motivate the search for complementary antigen markers for active TB [35].

In this study, we used a proteomic workflow to select MTB glycoproteins with predicted glycosylation and antigenic characteristics and used a purified glycoprotein preparation to develop a prototype competitive ELISA. Thirteen proteins were selected, and six showed compatibility values above 70%. The selection was based on computational predictions and therefore should be interpreted as candidate prioritization rather than experimental confirmation of glycosylation or immunogenicity for every individual protein.

The use of ConA affinity chromatography provided an enrichment step for glycoprotein-containing fractions before LC-MS identification. This approach has previously been used for mycobacterial glycoproteomics and can enrich mannose-containing glycoproteins [14]. The present study builds on our earlier work, which identified a larger set of candidate MTB glycoproteins [5], but the current analysis addresses a different question by using the purified glycoprotein preparation to develop a prototype antibody-based assay and to estimate its apparent performance in clinical specimens.

The immunochromatographic assay showed that the purified antibodies recognized TB-associated material in clinical specimens, with an apparent sensitivity of 92.4% and specificity of 94.6%. The subsequent competitive ELISA showed high apparent discriminatory performance, with an AUC of 0.951, sensitivity of 90.15%, and specificity of 93.24% at a cut-off of ≤0.65. These are apparent (internal) estimates, because the cut-off was selected and evaluated on the same 206 specimens, and they are therefore likely to be optimistic. They provide a preliminary basis for further analytical development of glycoprotein-based antigen detection but do not establish diagnostic accuracy; independent validation in larger and more diverse populations is needed.

Potential glycosylation sites in prokaryotic protein sequences were predicted using the freely available GlycoPP web server. Protein sequences in FASTA format were submitted for analysis, and predictions were reported with corresponding Support Vector Machine (SVM) scores, with higher scores indicating a greater likelihood of glycosylation.

The predicted glycosylation sites were compared with antigenic epitopes, and several proteins showed substantial overlap between glycosylated residues and antigenic regions. Thirteen proteins with high compatibility between predicted glycosylation sites and antigenic epitopes were selected, most of which were surface-exposed or membrane-associated proteins. Because glycosylation may influence the immunogenic properties of mycobacterial proteins and host–*Mycobacterium* interactions, these proteins were considered candidates for further evaluation as antigens [36], [37], [38], [39].

Recent studies have highlighted the important role of glycosylation in host–pathogen interactions, particularly in immune recognition. The survival of *Mycobacterium tuberculosis* depends on the coordinated activity of multiple bacillary components that modulate host immune responses. Among these, the multigene PE_PPE protein family includes several members implicated in immune evasion. Studies by Tiwari et al. suggest that the PE35–PPE68 gene pair contributes to immunomodulation and may influence the pathophysiology of TB [35], [40], [41]. In addition, Jung et al. showed that the purified 38-kDa glycolipoprotein of MTB H37Rv (PstS-1) activates the mitogen-activated protein kinase (MAPK) pathways, namely extracellular signal-regulated kinase 1/2 (ERK1/2) and p38, and induces the release of tumor necrosis factor-α (TNF-α) and interleukin-6 (IL-6) from human monocytes through Toll-like receptors 2 and 4 [42]. The 38-kDa glycolipoprotein of MTB H37Rv has also been shown to induce strong cellular and humoral immune responses in patients with TB [38].

Rv3873 was found to localize to the plasma membrane and induce the secretion of IL-10 and MCP-1 from THP-1 macrophages, while simultaneously decreasing the levels of IL-12p70. The induction of these cytokines was shown to be Toll-like receptor 2 (TLR2)-dependent when macrophages were stimulated with a combination of the PE35 and PPE68 proteins [41].

Rv0934 (PstS-1) has also been reported to induce interferon-γ (IFN-γ) and immunoglobulin IgG2a production; additionally, Rv0934 stimulates the deregulated production of IL-12 by macrophages and activates CD8^+^ cytotoxic T cells [43].

Rv0954 is a membrane-associated protein that localizes to the mid-cell in pre-divisional *M. tuberculosis* and subsequently tracks with the division process. Proper regulation of cell division is essential for bacterial growth, survival, pathogenesis, and antibiotic resistance. Wang et al. demonstrated that the previously uncharacterized Rv0954 protein specifically localizes to the division site and interacts with the cell division proteins LamA and PbpA, suggesting a role in coordinating bacterial cytokinesis [44].

Several MTB lipoproteins, including LpqH (19-kDa protein), LprA (Rv1270), and LprG (Rv1411c), are recognized by TLR-2. MTB lipopeptides can promote TLR-2-dependent maturation of dendritic cells and activation of naïve T cells, thereby contributing to the initiation of adaptive immune responses [45]. Additionally, Rv0341 is predicted to encode a glycine-rich cell wall protein that may contribute to cell wall stability. Notably, this gene is highly conserved within the MTBC but lacks orthologs in nonpathogenic *Mycobacterium* species, suggesting a potentially specific role in the biology of the complex [46].

A deeper understanding of MTB pathogenesis and the host factors it exploits to establish and maintain its intracellular niche is essential for developing more effective therapeutic strategies. Characterizing host immune responses and the mechanisms that regulate infection is particularly important, as these processes influence both disease progression and tissue pathology. Accordingly, increasing attention has been given to host-directed therapies that modulate immune responses to enhance mycobacterial clearance while limiting excessive inflammation and tissue damage. Such approaches may offer valuable complementary strategies for treating drug-resistant TB [6].

Arabinomannan (AM), a major carbohydrate antigen of MTB, is a component of lipoarabinomannan and has been investigated for TB vaccine development. Because of its structural complexity, synthetic AM analogues and AM–Ag85B conjugates have been explored as alternative vaccine strategies [7].

Although the role of glycosylation in antigen–antibody recognition remains incompletely understood, our findings suggest that glycosylation sites may preferentially occur near antigenic epitopes, potentially influencing immune recognition. Mycobacterial glycoproteins may therefore represent candidate targets for vaccines, antimicrobial therapies, and antigen-based assays, pending experimental validation. In addition, host immune biomarkers, including cytokines, IFN-γ responses, and antigen-specific antibodies, show potential for distinguishing active from latent TB and predicting disease progression and treatment response. Integrating these biomarkers with conventional diagnostic methods may improve TB detection and clinical management [47].

### 4.1 Limitations

This study has several limitations. First, the cut-off was selected and evaluated using the same 206 specimens, potentially overestimating diagnostic performance; no independent validation cohort or internal cross-validation was available. Second, the single-center design and absence of disease controls, including patients with nontuberculous mycobacterial infections and other respiratory diseases, limit the generalizability of the findings and leave specificity against these conditions undetermined. Third, antibodies were purified from pooled sera of only 10 patients, and the assay used a ConA-enriched glycoprotein fraction rather than individual recombinant proteins, preventing attribution of the diagnostic signal to a specific protein. Computationally predicted glycosylation sites and epitopes were not experimentally validated. Fourth, duplicate measurements were technical replicates, and independent replication of the complete assay was not documented. Finally, some methodological details relevant to reproducibility remain unavailable, namely the LC–MS/MS sequence database and version, its taxonomic scope, the peptide- and protein-level identification criteria, and the FDR threshold; the internal diameter of the LC column; inter-assay variability; and the detection limit. These limitations underscore the need for independent validation in multicenter cohorts that include appropriate respiratory disease controls, alongside further analytical characterization of the assay.

## 5 Conclusion

This study describes the enrichment and proteomic identification of MTB glycoproteins, the prioritization of candidates according to predicted glycosylation and epitope compatibility, and the development of a prototype antibody-based competitive ELISA for TB-associated antigen in sputum. In this single-center data set, the assay showed high apparent discriminatory performance (AUC 0.951), but these estimates were obtained on the same specimens used to select the cut-off and have not been independently validated. The glycoprotein candidates are immunologically relevant and warrant further investigation as antigen targets; whether they, or the assay described here, can contribute to TB diagnosis remains to be determined. Further validation in larger and diverse clinical populations, including disease-control groups, is required to determine the diagnostic accuracy, reproducibility, and feasibility of glycoprotein-based approaches.

## CRediT authorship contribution statement

**Shamsi Yari:** Writing – review & editing, Writing – original draft, Visualization, Validation, Supervision, Software, Resources, Project administration, Methodology, Investigation, Funding acquisition, Formal analysis, Conceptualization. **Seyed Davar Siadat:** Visualization, Investigation, Formal analysis. **Fatemeh Yari:** Visualization, Investigation, Formal analysis. **Abolfazl Fateh:** Writing – review & editing, Visualization, Validation, Methodology, Investigation, Formal analysis. **Alireza Hadizadeh Tasbiti:** Writing – review & editing, Writing – original draft, Supervision, Software, Resources, Project administration, Funding acquisition, Conceptualization.

## Ethics declaration

Written informed consent to take part in the study and to publish the article has been obtained from all participants or their legal representatives. The privacy rights of participants have been observed.

This study included organ or tissue donors. This study includes human biological material and consent was obtained by donors, or their next of kin or legal representatives, for use in this study and for publication of the article. The samples used in this research were not sourced from executed prisoners or prisoners of conscience.

This study was performed in compliance with relevant laws, regulatory frameworks and guidelines where the research took place. This study was approved by the Pasteur Institute of Iran. (Approval No. IR.PII.REC.1401.029)

## Declaration of generative AI and AI-assisted technologies in the manuscript preparation process

During the preparation of this work, the authors used ChatGPT (OpenAI) to improve the readability and language of the manuscript. After using this tool, the authors reviewed and edited the content as needed and take full responsibility for the content of the published article. No generative AI tool was used to generate, alter, or interpret the experimental data or research results.

## Declaration of competing interest

The authors declare that they have no known competing financial interests or personal relationships that could have appeared to influence the work reported in this paper.

## Data Availability

The data supporting the findings of this study are provided in the article and its supplementary material (Table 1, Table 2, Table 3 and Table S1). The de-identified, specimen-level ELISA optical density values and immunochromatographic results underlying the ROC curve and diagnostic accuracy analyses are available from the corresponding authors upon reasonable request. The LC–MS/MS raw data are available upon request from the corresponding authors. The purified antibodies and HRP–glycoprotein conjugate are also available from the corresponding authors upon reasonable request, subject to applicable institutional material transfer procedures.
