# Supplementary material for: Proteomic identification of Mycobacterium tuberculosis glycoproteins as potential biomarkers for active tuberculosis: Development of a prototype competitive ELISA for sputum antigen detection
Source: Biochem Biophys Rep. 2026 Nov 5;48:102867. doi: 10.1016/j.bbrep.2026.102867 (PMC13753359; doi:10.1016/j.bbrep.2026.102867)
Supplement: Supplementary file 1 [file mmc1.docx]

**Supplementary Table 1*:*** *Mycobacterium tuberculosis* glycoproteins were identified by Affinity Chromatography-Con A (Concanavalin A) followed by Mass spectrometry and Predicted Linear Epitope peptides by immune epitope database (IEDB)

| **protein** | **Protein length –function category^1^** | **O-glycol (number-%)^2^** | **MW – PI^3^** | **Predicted Linear Epitope peptides based on residue scores (Bepipred Linear Epitope Prediction 2.0 Results)^4^** |
| --- | --- | --- | --- | --- |
| **Rv3873** | 368 amino acids  Length  PE/PPE | O-glycol (43 , 11.68%): 33, 35, 51, 57, 61, 69, 79, 80, 84, 90, 97, 102, 115, 120, 122, 129, 172, 179, 182, 183, 191, 194, 195. 196, 206, 216, 230, 231, 234, 239, 257, 258, 261, 269, 272, 282, 293, 297, 316, 317, 319, 336, 339 | 37330.0 Da  3.9857 | EAWTG, FEKLEPMASILDPGASQSTTNPIFGMPSPGSSTPVGQLPPAATQTLGQLGEMSGPMQQLTQPLQQVTSLFSQVGGTGGGNPADEEA, HPLAGGSGPSAGAGLLRAESLPGAGG, SQLIEKPVAPSVMPAAAAGSSATGGAAPVGAGAMGQGAQSGGSTRPGLVAPAPLAQEREEDDEDDWDEE |
| **Rv0934** | 374 amino acids  cell wall and cell processes | O-glycol(34 , 9.09%): 26, 30, 32, 35, 41, 44, 45, 48, 49, 52, 56, 59, 80, 82, 86, 88, 128, 186, 192, 210, 215, 216, 236, 241, 269, 280, 290, 292, 299, 328, 341, 347, 373, 374 | 38211.1 Da  5.0223 | GSKPPSGSPETGAGAGTVATTPASSPV, GSG, YLSEGDMAAHK, GVSEHLKLNG, QGTIKTWDDPQIAALNPGVNLP, S, SKQDPEGWGKSPGFGTTVDFPAVPGALGENGNGG, QASQRGL, SSGNFLLP, AAGFASKTPANQAISMIDGPAPD, RQKDAATAQ, NKASFLDQV, FQPL,PA, VK |
| **Rv1078** | 240 amino acids  conserved hypotheticals | O-glycol(23 , 9.58%): 10, 21, 35, 44, 45, 47, 67, 87, 89, 122, 123, 126, 127, 139, 142, 170, 173, 174, 178, 186, 188, 189, 234 | 25076.0 Da  7.2792 | MTEQPPPGGSYPPPPPPPGPSGGHEPPPAAPPGGSGYAPPPPPSSGSGYPPPPPPPGGGAYPPPPPSAGGYAPPPPGPAIRTMPTESYTP, S, EY, YRQGTIGSS, ETTGQPIG, QT |
| **Rv0954** | 303 amino acids  cell wall and cell processes | O-glycol(42 , 13.86%): 4, 18, 23, 34, 55, 63, 74, 77, 101, 119, 126, 129, 130, 156, 160, 204, 210, 218, 224, 225, 226, 228, 230, 232,235, 246, 248, 251, 256, 257, 260, 264, 266, 272, 275, 277, 281, 287, 290, 295, 296, 297 | 30203.7 Da  6.7938 | MTYSPGNPGYPQAQPAGSYGGVTPSFAHADEGASK, ELGGGDGAVSGDTGLP, K, FNKPSAYST |
| **Rv0173** | 390 amino acids  cell wall and cell processes | O-glycol(21 , 5.38%):29, 30, 37, 47, 53, 61, 68, 79, 107, 114, 146, 150, 151, 182, 215, 255, 262, 270, 334, 342, 347 | 42239.6 Da  8.4365 | ISNVAIPGGPGTGPG , AING, IKL, TL, SLLG, DPSPVPLKDGDTIPLKRSSAYPTTEQTLA, LRGGGLVNLEG, Q, AD, NQQRDDITRAIDSTNR, PPLIKHFAD, QGRLSQS, DQY,S,G,Q,Q, R, FDVDTVPQLVRG, SAIDNAFLTGTGFSGALRALEQSFGRDPETMIPDIRYTPNPNDAPGGPLVERGN \| |
| **Rv0583c** | 228 amino acids  cell wall and cell processes | O-glycol(27 , 11.84%): 5, 10, 14, 28, 31, 32, 33, 35, 36, 38, 40, 41, 42,43, 44, 45, 46, 47, 49, 50, 51, 154, 158, 166, 173, 192, 225 | 23682.4 Da  4.386 | FNIKTDSAPTTSPTTTSPTTSTTTTSATTSAQAAGPNY, HIQETPVHHGDPG, PDDWRLLPESSRA, PADPN, PGELKNLPGFQGSGDGSAATLGGFS, SKNGKLR, DDETMTLMD |
| **Rv1270** | 244 amino acids  cell wall and cell processes | O-glycol(25 , 10.25%): 13, 26, 35, 37, 40, 42, 61, 94, 96, 98, 105, 135, 164, 172, 176, 179, 180, 186, 194, 202, 208, 211, 212, 222, | 24874.0 Da  5.2108 | TEGDAGKASDTAATASNGD, D, MRKV, DVPNLRVTKLEGDISNTPQTV, GNKSED, DLGQPGTYTDFGNGASIYNV, D, NKG, DASVAGSQQA, SSADDIATLAGSRLTSEDVK, SS, T, DWGKQVT |
| **Rv1543** | 341 amino acids  lipid metabolism | O-glycol(21 , 6.16%):18, 23, 26, 59, 62, 63, 106, 115, 193, 199, 210, 242, 246, 256, 267, 278, 279, 310, 311, 318, 329 | 36820.9 Da  9.3091 | NF, AA, SNIVNTPNSAGRY, DAVQGRNLNDAV, ENLENV, N, RAIRGN, D, IRRSLELSYDRIHDYQ, TRA, SPTTIYDKFPTLTP ,PRRASS, FNMFGDSSAAKGSESQTDTSELDKRSETFVRATRG |
| **Rv0341** | 479 amino acids  cell wall and cell processes | O-glycol(34 , 7.1%): 3, 14, 20, 30, 31, 43, 62, 93, 96, 97, 105, 122, 123, 141, 160, 189, 232, 237, 249, 252, 255, 256, 262, 265, 273, 287, 316, 345, 385, 393, 404, 420, 429, 470 | 43921.2 Da  5.3709 | DAAR, VAA, GRAMT, I, NLGAGDPMSG, GFAGDAGAG, GAG, A, L, SSGGFGGQ, G, GAQ, G, G, GLGAQ, GMGFG, G, GLGLGGQAGGVIGGSAAGAIGAGVGGRLGGNGQIGVAGQGAVGAGVGAGVGGQAGIA, GVSAGGGLGGVGNVSGLTGV, NASGQ, GLI, GAALN, MPHL, GPLAGVGVGGQAGAAGGAGL, VGHPTPQPAALG, VVAKTEAAAGVVGGVGGATAAGVGGAHG, IL, AALG, VDTVNAGVTPV, LIHGGTGGYGGMNPPVTDAPAPQVPARAQPMTTAAEHTPAVTQPQHTPVEPPVHDKPPSHSVFDVGHEPPVTHTPPAPIEL |
| **Rv1860** | 325 amino acids  cell wall and cell processes | O-glycol(21 , 6.46%): 25, 30, 49, 50, 53, 56, 57, 66, 131, 146, 147, 177, 183, 211, 218, 232, 240, 245, 282, 315, 316 | 32720.8 Da  4.7048 | QVDPNLTR, PATANADPEPAPPVPTTAASPPSTAAAPPAPATPVAPPPPAAANTPNAQPGDPNAAPPPADPNAPPPPVIAPNAPQPVRIDNPVG, GWVES, AAHF, KTTGDPPFPGQPPPVAND, Y, SAEATDSKAAARLGS, MPYPGTRINQ, VSLDANGVSGSASY,KFSDPSKPNGQI,IGSPAANAPDAGPPQ,ANNPVDKGAAKA |
| **Rv2560** | 325 amino acids  cell wall and cell processes | O-glycol(24 , 7.38%): 2, 23, 44, 76, 77, 80, 86, 98, 125, 130, 135, 136, 139, 140, 142, 146, 203, 240, 246, 250, 256, 261, 264, 302 | 33124.4 Da  6.0752 | MSQPPEHPGNPADPQGGNQGAGSYPPPGYGAPPPPPGYGPPPGTYLPPGYNAPPPPPGYGPPPGPPPPGYPTHLQS, FSV, A, RATTAYTNTSGVSSESVDITMT, ADGKPV, DRSTSPIDSVKA, ETVGSNIG, L |
| **Rv0174** | 515 amino acids  virulence, detoxification, adaptation | O-glycol(32 , 6.21%): 17, 33, 49, 64, 69, 86, 102, 114, 122, 123, 128, 134, 179, 207, 211, 231, 238, 246, 255, 260, 298, 301, 303, 333, 337, 344, 425, 454, 472, 473, 476, 480, 483, 500, 501, 502, 515 | 54005.4 Da  5.5061 | DLPASGGLYPTANV, TAVEPTDQGAR, KIPVDASAN, AV, TGAPGKYFSSGQTITKGTVPSEIGPALDNSNR, L, AQAVGGLGP, A, TNIG, DIIEN, GPILDSQVNTGDQIE, AQTATRDQN, QAAPTADEV, DSLPQ, AI, LTPTPGAAQLP, AINYPPPC, SEWRSPADTSPRPLPSGTYCKIPQD, QLQ, RAATPKECRSKDPYVPLGTNPWFGDPNQILTCPAPGARCDQPVKPGLVI, PSINTGLNPAPADQVQGTPPPVSDPLQRPGSGTVQCNGQQPNPCVYTPTSGPSAVYSPASGELVGPDGVKYAVANSSTTGDDGWKE, A |
| **Rv0838** | 256 amino acids  cell wall and cell processes | O-glycol(20 , 7.81%): 27, 32, 36, 38, 39, 47, 49, 51, 58, 84, 89, 158, 164, 168, 171, 178, 206, 210, 230, 233 | 27288.9 Da  7.1368 | VSAGRWSESPSATSWPVRPVNTTTPSGPVPPVSEAARAAGLVDVRGV,NNFTGTQLYPPGA,VVPNPAWVARPGKYAHSHE,AQRQCPSVRRSGELCLA,FDDFSSRATAFATQG,SA,LTVYSG,FDGPGAGVDRPIL |

**1.** Protein length is calculated in amino acids (aa), based on the coding sequences in the genome

**2**. O-linked glycosylation sites

**3.** Molecular weight, kilo Dalton (KDa); the isoelectric point (pI) of a protein is defined as the pH

**4.** The results showing that this dataset BepiPred-2.0 outperforms based on residue scores obtained from the immune epitope database <http://www.iedb.org>
